# Noninvasive oxygenation and ventilation strategies for viral acute respiratory failure: a comprehensive systematic review and meta-analysis

**DOI:** 10.1186/s13643-025-02775-6

**Published:** 2025-02-04

**Authors:** Fredy Leonardo Carreño-Hernández, Sergio Prieto, Daniela Abondando, Jairo Alejandro Gaitán, Yenny Rocío Cárdenas -Bolívar, Adriana Beltrán, Jorge Iván Alvarado-Sánchez, Joseph L. Nates

**Affiliations:** 1 Clinical Research, School of Medicine, Universidad de los Andes, Bogotá, D.C. Colombia; 2 School of Medicine, Universidad de los Andes, Bogotá, D.C. Colombia; 3 Clinical Research, Neurology, Hospital Universitario Fundación Santa Fe de Bogotá, Bogotá, Colombia; 4 Intensive Care Unit director, Hospital Universitario Fundación Santa Fe de Bogotá, Bogotá, Colombia; 5 Epidemiology Research, Bogotá, Colombia; 6 Hospital Universitario Fundación Santa Fe de Bogotá, Intensive Care Unit, Universidad Nacional de Colombia, Bogotá, Colombia; 7 Division of Anesthesiology, Critical Care, and Pain Medicine, The University of Texas MD Anderson Cancer Center, Texas, Houston, TX U.S.

**Keywords:** Critical care, Mechanical ventilation, Noninvasive ventilation, COVID-19, SARS-CoV-2

## Abstract

**Background:**

The COVID-19 pandemic has resulted in a critical shortage of respiratory ventilators, highlighting the urgent need to explore alternative treatment options for patients with acute respiratory distress syndrome (ARDS) caused by respiratory viruses, as an alternative to invasive mechanical ventilation (IMV) in future pandemics.

**Objectives:**

The objective of this study was to assess the effectiveness of alternative noninvasive oxygenation and ventilation strategies in comparison to invasive mechanical ventilation (IMV) in patients with virus-induced acute respiratory failure (ARF). The primary outcome was the all-cause ICU mortality rate.

**Methods:**

A systematic review was conducted following the Cochrane guidelines and PRISMA reporting guidelines. The search encompassed databases such as Medline, Cochrane CENTRAL, and Embase to identify relevant indexed literature. Additionally, gray literature was included by consulting regulatory agencies. The included studies compared various oxygenation and ventilatory alternatives, such as high-flow nasal cannula (HFNC), continuous positive airway pressure (CPAP), or noninvasive mechanical ventilation (NIMV) with IMV. An exploratory meta-analysis was performed by calculating the risk ratio (RR) by random effects and meta-regression to explore possible sources of heterogeneity and to compare ventilatory alternatives against IMV to reduce mortality, length of stay (LOS) days in ICU, nosocomial infection, and barotrauma.

**Results:**

A total of forty-seven studies were included in this systematic review. NIMV had an RR of 0.70 (0.58–0.85), HFNC had an RR of 0.54 (0.42–0.71), and CPAP had an RR of 0.80 (0.71–0.90), with meta-regression models that reduced heterogeneity to 0%. For LOS days in ICU, NIMV had 0.38 (− 0.69: − 0.08) lower days and HFNC 0.29 (− 0.64: 0.06) lower days with meta-regression models that reduction heterogeneity to 0% for HFNC and 50% for NIMV. Not enough studies reported nosocomial infection or barotrauma to evaluate them in a meta-analysis. The overall quality of evidence, as assessed by GRADE evaluation, was determined to be from very low to medium certainty depending on the ventilatory strategy and outcome.

**Conclusions:**

The findings of this systematic review support the use of alternative noninvasive oxygenation and ventilation strategies as viable alternatives to conventional respiratory ventilation for managing viral-induced ARF. Although it is essential to interpret these findings with caution given the overall low to medium certainty of the evidence, the integration of these modalities as part of the management strategies of these patients could help reduce the utilization of ICU beds, invasive ventilators, and costs in both developed and developing countries.

**Supplementary Information:**

The online version contains supplementary material available at 10.1186/s13643-025-02775-6.

## Introduction

The COVID-19 pandemic has brought forth unprecedented challenges in the management of critically ill patients, especially within the confines of intensive care units (ICUs), where the provision of ventilatory support holds pivotal importance [1]. Among these patients, acute respiratory failure (ARF) resulting from COVID-19 and other viral infections is the leading cause of mortality [2]. Additionally, the global shortage of respiratory ventilators, as underscored by the World Health Organization (WHO), has further intensified the severity of the situation [2]. The elevated mortality rates observed among ICU patients requiring ventilatory support emphasize the pressing need for effective interventions [3].

Numerous ventilatory strategies have been described to address the medical management of ARF, including noninvasive ventilation (NIV), invasive mechanical ventilation (IMV), and standard oxygen therapy (SOT). Notably, compared with SOT and IMV, NIV has been shown to decrease mortality in patients with acute respiratory distress syndrome (ARDS), a form of ARF [4]. Furthermore, the utilization of NIV in COVID-19 patients has shown promise in reducing mortality [5]. A systematic review provided evidence supporting the efficacy of NIV over IMV in managing acute hypoxic respiratory failure caused by coronaviruses, such as COVID-19, severe acute respiratory syndrome (SARS), and Middle East respiratory syndrome (MERS). However, that review was limited in scope, as it did not encompass patients with ARDS caused by H1N1 [5].

To address these gaps and contribute to the understanding of optimal oxygenation and ventilation strategies, this study aimed to comprehensively evaluate the efficacy and safety of alternative techniques. These included HFNC therapy, CPAP therapy, NIV, ventilator splitters (VS), and low-cost ventilators (LCVs) for the management of ARF caused by SARS-CoV-2, SARS, MERS, or H1N1. The primary outcome measure was all-cause ICU mortality, with a specific focus on mortality in cases of NIV failure, serving as a crucial safety indicator.

## Methods

### Protocol

We conducted a systematic review adhering to the Preferred Reporting Items for Systematic Reviews and Meta-Analyses (PRISMA) framework [6] as a reporting guideline and followed the guidelines outlined in the Cochrane Handbook for Systematic Reviews of Interventions [7]. The study protocol was registered at PROSPERO (CRD42020199175).

### Eligibility criteria

Randomized clinical trials (RCTs), nonrandomized studies (NRSs) (cohorts and case‒controls), or congress abstracts, if they had results, were included. The inclusion criterion for identifying both published and unpublished studies was abstracts from congresses, which were indexed by the authors’ names. The study followed the PICO framework, which is structured as follows:

Patients (P): This study concentrated on critically ill adult patients diagnosed with ARF attributed to SARS-CoV-2, SARS, MERS, or H1N1 infections. The inclusion criterion was patients who were monitored until ICU discharge, irrespective of their final outcome. Virus identification relies on diverse methods, including polymerase chain reaction (PCR), antigen testing, or epidemiological association. ARDS was defined in accordance with the Berlin criteria.

Intervention (I): The intervention included an array of respiratory support methods, including high flow nasal cannula (HFNC), continuous positive airway pressure (CPAP), NIMV, and invasive strategies such as ventilator splitters or LCV. The following specific definitions were established for each method:

- HFNC: Continuous flow at or exceeding 10 ml/min, alongside specified temperature and humidification parameters.
- CPAP: Continuous pressure equal to or exceeding 5 mmHg delivered via a facemask.
- LCV: Mechanical compression of resuscitation bags.
- Ventilator splitters (VS): Devices with or without resistance valves.
- NIMV: Ventilation using masks or helmets, encompassing noninvasive positive-pressure ventilation or bilevel positive airway pressure (BiPAP). These precise definitions ensured consistency and clarity in describing the distinct respiratory support methods utilized in the study.

Comparator (C): The comparator used was invasive mechanical ventilation (IMV).

Outcome (O): The primary outcome of interest was all-cause ICU mortality. Secondary outcomes where length of stay in ICU (LOS), barotrauma, and nosocomial infection.

### Exclusion criteria

The researchers excluded patients from registers that involved ARF caused by other factors, cases where the viral infection was not confirmed, case series, case reports, consensus papers, or articles written in languages other than Spanish or English.

### Search strategy

The comprehensive search strategy for this systematic review can be found in the PROSPERO protocol with the registration code CRD42020199175. The EMBASE, MEDLINE, and Cochrane CENTRAL databases were searched between January 2000 and August 2021 and between September 2021 and December 2022. The retrieved articles were downloaded and subjected to manual selection using the Rayyan™ platform to identify relevant indexed literature.

To include gray literature, additional research was performed on regulatory and governmental agency websites such as clinicaltrial.gov, the Food and Drug Administration (FDA), and the “Instituto Nacional de Vigilancia de Medicamentos y Alimentos (INVIMA)”. For studies on trials, manual selection was conducted based on the engineered searches, as the downloadable content was not compatible with the Rayyan™ platform.

### Study selection

In the research process, three authors (C.F.L., G.J.A., and A.D.) reviewed the abstracts based on the predefined inclusion criteria. Afterward, two authors (C.F.L. and P.S.) independently reviewed the full texts of the selected articles. Studies that met the inclusion criteria were included in the data extraction process. In case of any discrepancies or disagreements, the authors resolved them through consensus among themselves.

### Extracted data

A standardized data table form in Microsoft Excel was developed to include all the data, and two authors extracted the information (C.F.L.; A.D.). The extracted data included country, study design, year, virus, intervention at admission, total patients, patients in the comparator, patients on intervention with subsequent intubation, ventilatory parameters, PaO_2_/FiO_2_, rate oxygenation (ROX) index, APACHE II score, smoking habit, hypertension, ARDS classification, age, antiviral and anti-inflammatory treatment, and outcomes such as mortality in the ICU, LOS ICU, barotrauma, and nosocomial infection. Continuous variables are reported as medians or medians with their respective standard deviations or interquartile ranges, respectively, while categorical variables are reported as frequencies and percentages. Incomplete data were registered as “no data” or extrapolated from the frequency of patients in interventions and IMV if possible. For studies in trials, the extracted data included the study reference, country, study design, intervention, and phase.

### Risk of bias in individual studies

Two independent authors (C.F.L.; P.S.) assessed the risk of bias using the Cochrane Risk of Bias (RoB) tool for randomized controlled trials (RCTs) or the Risk of Bias in Nonrandomized Studies of Interventions (ROBINS-I) tool for nonrandomized studies (NRS) [8–10]. For RoB, it was evaluated the random sequence generation, the allocation concealment, the binding of participants and personnel, the blinding of outcomes, incomplete outcome data, and selective reporting. For ROBINS-I bias for confounding, in selection of participants in the study, in classification of interventions, in deviations from intended interventions, due to missing data, in measurement of outcomes and in selection of the reported results were evaluated. No bias assessment was conducted for abstracts presented at congresses or trials on course due to incomplete information.

### Synthesis of evidence (meta-analyses)

Meta-analysis was conducted using a random effects model for each ventilatory strategy. For dichotomous outcome RR was calculated and mean differences for continues outcomes were obtained, both with confidence intervals 95% (CIs) calculated. One analysis was performed combining all RcT and NRS, while other analysis only between same studies design was performed. Heterogeneity was evaluated by the Cochran Q statistic, and its effect was measured using inconsistency [*I*^2^] [10]. Sensitivity analysis was performed by excluding studies with high risk of bias to evaluate the impact of bias in results. Asymmetry was visually evaluated by funnel plots and by Egger’s test for each intervention with and without abstract congress for publication bias evaluation. The data were analyzed using R version 4.2.2 with meta-packages, and significance level of *P* < 0.05 was considered to indicate statistical significance.

### Subgroup analyses and heterogeneity evaluation (meta-regression analysis)

The meta-regression analysis included age, PaO_2_/FiO_2_, virus type, SOFA score, and APACHE II score means for interventions. Studies reporting medians with interquartile ranges or maximum and minimum values were transformed into means with standard deviations using Sean McGrath’s Box‒Cox method. Incomplete data were imputed out (generated) using the "mice" package and the "cart" method so missing data can be filled. The sensitivity analysis involved various combinations of included or excluded abstracts, with or without imputed data and with only those studies that had the lowest risk of bias. The chosen models were based on covariate combinations with the lowest heterogeneity.

### Quality of evidence

To evaluate the quality of evidence and formulate clinical recommendations, the researchers employed the Grading of Recommendations, Assessment, Development, and Evaluations (GRADE) approach [11, 12]. The components evaluated in GRADE were the study design, risk of bias, inconsistency, indirectness, imprecision, and other considerations. To facilitate the grading process and create evidence profiles, the researchers utilized GRADEpro™ software.

## Results

### Study selection

**Fig. 1:**
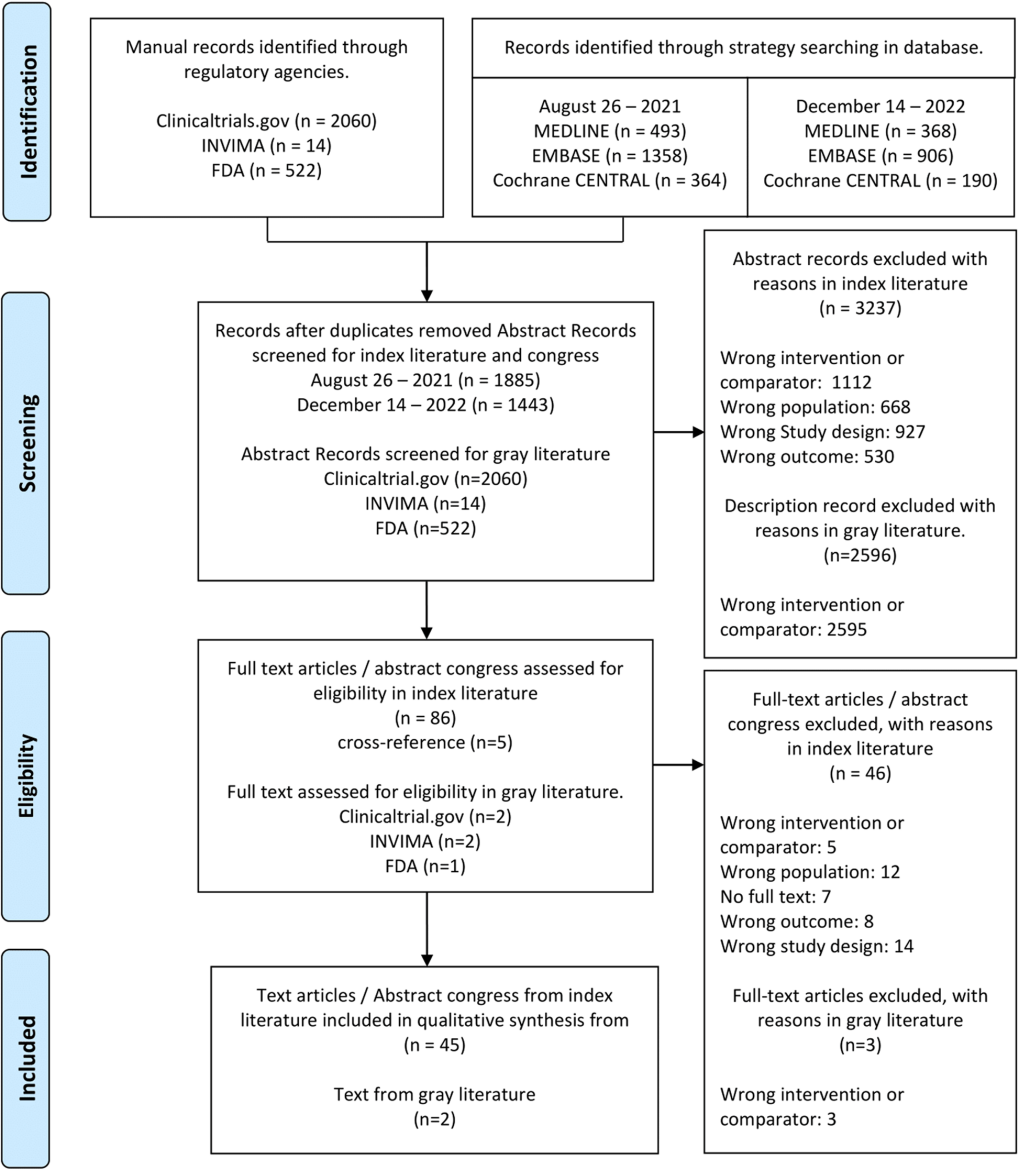
PRISMA flowgram. FDA: Food and Drug Administration. INVIMA: Instituto Nacional de Vigilancia de Medicamentos y Alimentos

A total of 5739 records were initially identified through the literature search. After applying the inclusion and exclusion criteria, 47 studies were included in the review (Fig. 1). Of these, 36 were nonrandomized studies (NRS) [13–49], 2 were trial studies [50, 51], and 9 were abstracts from congresses [52–60]. As of December 2023, no published articles derived from the Abstract congresses included were identified.

### Study characteristics

The study included 36 NRS and nine abstract congress studies. Among these, 12 studies concentrated on NIMV [15, 18, 20, 21, 23, 26–29, 40, 46, 60], seven on CPAP [19, 33, 41, 44, 55, 57, 59], and six on HFNC [25, 32, 36, 39, 47, 53]. Five studies evaluated multiple interventions, four involving NIMV and HFNC [13, 14, 30, 60] and one involving NIMV and CPAP [34]. Fifteen studies did not distinctly differentiate between interventions. Among the 36 NRS studies, 16 were prospective cohort studies [13–15, 17–21, 23–28, 41, 49], 19 were retrospective cohort studies [22, 29–46], and one was a nested case‒control study [48]. Various clinical characteristics were reported across the studies, including ventilatory parameters, age, frequency of hypertension, ICU days, SOFA score, APACHE II score, PaO_2_/FiO_2_ levels, anti-inflammatory and antiviral treatments, smoking habits, ARDS classification, barotrauma, and nosocomial superinfection. Additionally, ongoing trials related to noninvasive ventilation (NIV) and spontaneous ventilation (SV) have been conducted with no results published. Detailed information on each study’s characteristics and evaluated variables can be found in Table 1 and the online supplement (Table S1).

**Table 1:** Studies general characteristics

Author	Design County	Virus	Interventions Population	Ventilatory parameters	APACHE-II Score	SOFA score	AGE	PaO_2_/FiO_2_	RR [IC95%]
1. Asghar 2020 [17]	Prospective cohort Pakistan	SARS-CoV-2	IMV: 50 CPAP: 59	No data	No data	No data	No data	No data	- 0.60 [0.45, 0.80]
2. Bertaina 2020 [18]	Prospective cohort Multicountry	SARS-CoV-2	IMV: 106 NIMV: 390	No data	No data	No data	67 (57–73) 70 (58–79)	No data	- 0.49 [0.42, 0.58]
3. Brink 2012 [19]	Prospective cohort Sweden	H1N1	IMV: 26 CPAP: 67	FiO_2_: 90% PEEP: 5 TDV: 8.9	No data	No data	52 (38–58) 49 (38–59)	80 (40–108) 86 (63–116)	- 2.20 [0.70, 6.88]
4. Garcia 2021 [13]	Prospective cohort Switzerland	SARS-CoV-2	IMV: 92 NIMV: 87 NIMV-F: 43 HFNC: 87 HFNC-F: 45	FiO_2_: 63% PEEP: 12 TDV: 6.1 FiO_2_: 41% PEEP: 12 TDV: 6.1 FiO_2_: 60%	11 (8–20) 10 (7–16) No data 10 (6–13) No data	7 (6–8) 6 (4–7) No data 6 (3–7) No data	62 (55–70) 66 (55–76) No data 63 (55–74) No data	123 (90–165) 135 (97–168) No data 126 (79–169) No data	- 1.47 [0.94, 2.30] 1.49 [0.88, 2.52] 0.78 [0.45, 1.36] 1.24 [0.71, 2.18]
5. Masclans 2013 [20]	Prospective cohort Spain	H1N1	IMV: 312 NIMV: 177 NIMV-F: 105	No data	15 (10,–19) 12 (9–16) 14 (10–18.2)	No data	43 (32–52) 44 (33–53) 44 (32–53)	No data	- 0.66 [0.44, 0.97] 1.11 [0.76, 1.61]
6. Mellado-Artigas 2021 [49]	Prospective cohort Spain	SARS-CoV-2	IMV (A): 61 IMV (B): 251 HFNC (A): 61 HFNC-F (A): 21 HFNC (B): 95	FiO_2_: 79% O_2_ L/min: 55 FiO_2_: 72%	11 (9–14) 12 (10–14) 10 (9–113) No data 10 (7–13)	5 [3,–7] 7 [5–8] 4 [4–7] No data 4 [3–5]	61 (11) 62.3 (10.9) 62 (11) No data 60.6 (12.7)	117 (51) 121.2 (58.0) 121 (49) No data 122.7 (48.5)	- - 0.69 [0.32, 1.50] 1.34 [0.58, 3.08] 0.34 [0.19, 0.60]
7. Mukhtar 2020 [21]	Prospective cohort Egypt	SARS-CoV-2	IMV: 9 NIMV: 30	No data	10 ± 4 11 ± 5.5	No data	No data	175 (118–205) 170 (112–224)	- 0.13 [0.04, 0.40]
8. Polok 2022 [23]	Prospective cohort Multicountry	SARS-CoV-2	IMV: 1876 NIMV: 630	No data	No data	7 (4–8) 4 (3–6)	75.1 ± 4.2 76.8 ± 4.9	No data	- 1.01 [0.92, 1.11]
9. Rama-Maceiras 2021 [24]	Prospective cohort Spain	SARS-CoV-2	IMV: 123 NIV: 214 NIV-F: 110	No data	No data	No data	No data	No data	- 0.46 [0.29, 0.74] 0.90 [0.57, 1.43]
10. Rello 2012 [25]	Prospective cohort Spain	H1N1	IMV: 10 HFNC: 20 HFNC-F: 10	O_2_ L/min: 25 O_2_ L/min: 30	25 (13–31) 11 (8–14) No data	8 (4–12) 3 (3–5) No data	50 (44–56) 37 (29–47) No data	73 (56–81) 135 (84–210) No data	- 0.75 [0.15, 3.79] 1.50 [0.32, 7.14]
11. Reyes 2022 [14]	Prospective cohort Multicountry	SARS-CoV-2	IMV: 14,197 NIMV: 24,112 NIMV-F: 6749 HFNC: 28,256 HFNC-F: 3538	No data	No data	No data	No data	No data	- 1.03 [1.00, 1.05] 1.30 [1.26, 1.35] 0.91 [0.89, 0.94] 1.13 [1.09, 1.19]
12. Ríos 2009 [26]	Prospective cohort Argentina	H1N1	IMV: 129 NIMV: 49	FiO_2_: 80% ^a^ PEEP: 10 ^a^ TDV: 7.8 ^a^ PP: 26 ^a^	19 ± 7 16 ± 6	No data	44 ± 15 40 ± 16	129 (70 to 173) 141 (92 to 217)	- 0.76 [0.51, 1.12]
13. Rodríguez 2017 [15]	Prospective cohort Spain	Influenza	IMV: 1092 NIMV: 806 NIMV-F: 458	No data	7 (5–10) 7 (4–9) 4 (3–6)	7 (5–10) 7 (4–9) 4 (3–6)	51 (39–62) 58 (47–68) 53 (42–65)	No data	- 0.78 [0.68, 0.91] 1.23 [1.06, 1.42]
14. Sivaloganathan 2020 [27]	Prospective cohort UK	SARS-CoV-2	IMV: 21 NIMV: 82 NIMV-F: 27	No data	22 (15–25) 11 (8–12.5) 18 (13.0–24.5)	6 (4–8) 3 (4–3) 4 (3–6)	61 (18–65) 50 (45–60) 57 (50–64)	No data	- 0.98 [0.46, 2.10] 0.39 [0.11, 1.38]
15. Yam 2005 [28]	Prospective cohort China	SARS-CoV1	IMV: 451 NIMV: 42	No data	No data	No data	44 47	No data	- 0.29 [0.09, 0.86]
16. Alraddadi 2019 [29]	Retrospective cohort Saudi Arabian	MERS	IMV: 197 NIMV: 105 NIMV-F: 97	No data	No data	7 (4–9) 9 (7–12) 7 (4–9)	58 (45–69) 60 (50–73) 61.0 (52–73)	106 (68, 166) 110 (62, 160) 103 (62, 160)	- 0.88 [0.75, 1.02] 0.95 [0.82, 1.10]
17. Baqi 2021 [30]	Retrospective cohort Pakistan	SARS-CoV-2	IMV: 57 NIMV: 100 HFNC: 21	No data	No data	No data	60 (50–65)	No data	- 0.77 [0.67, 0.89] 0.51 [0.33, 0.81]
18. Berenguer 2020 [31]	Retrospective cohort Spain	SARS-CoV-2	IMV: 619 NIV: 528	No data	No data	No data	70 ^a^	No data	- 1.00 [0.88, 1.14]
19. Castro 2022 [32]	Retrospective cohort Spain	SARS-CoV-2	IMV: 101 HFNC: 84 HFNC-F: 52	No data	No data	No data	127 ± 55 148 ± 64 No data	64 ± 14 66 ± 12 No data	- 0.55 [0.36, 0.84] 0.90 [0.49, 1.63]
20. Domenico 2021 [33]	Retrospective cohort Italy	SARS-CoV-2	IMV: 13 CPAP: 90 CPAP-F: 36	No data	No data	No data	56.231 ± 3.44 58.444 ± 2.79 No data	194.09 ± 29.81 247.87 ± 16.73 202.82 ± 16.88	- 0.69 [0.49, 0.99] 0.61 [0.39, 0.97]
21. Duca 2020 [34]	Retrospective cohort Italy	SARS-CoV-2	IMV: 7 NIMV: 7 CPAP: 71	No data	No data	No data	64 (62–72) 72(59–80) 70 (62–79)	76 (60–177) 87 (53–120) 131 (97—190)	- 0.60 [0.33, 1.08] 0.76 [0.67, 0.87]
22. Forrest 2021 [35]	Retrospective cohort Germany	SARS-CoV-2	IMV:154 NIV: 534	No data	No data	No data	68 ± 13 67 ± 16	No data	- 0.38 [0.33, 0.44]
23. Hernandez 2020 [36]	Retrospective cohort USA	SARS-CoV-2	IMV: 97 HFNC: 109 HFNC-F: 78	No data	No data	No data 7 (5–10) 9 (7–11)	No data 62 (55–73) 65 (56–75)	148 (111–205) ^a^	- 0.55 [0.36, 0.84] 0.77 [0.51, 1.16]
24. Hesselle 2022 [37]	Retrospective cohort Germany	SARS-CoV-2	IMV: 492 NIV: 87	No data	No data	No data	No data	No data	- 0.95 [0.71, 1.26]
25. Hua 2020 [38]	Retrospective cohort China	SARS-CoV-2	IMV: 113 NIV: 152	No data	No data	6.0 ± 3.0 5.5 ± 2.7	67 ± 13 71 ± 10	No data	- 0.44 [0.36, 0.54]
26. Lee 2020 [39]	Retrospective cohort South Korea	SARS-CoV-2	IMV: 23 HFNC: 24 HFNC-F: 16	No data	15 (10–17) 11 (8–14) 14 (8–15)	3 (2–7) 2 (2–4) 3 (2–4)	72 (64–76) 69 (60–78) 66 (59–77)	86 (69–123) 144 (70–206) 120 (62–188)	- 0.59 [0.30, 1.15] 0.77 [0.40, 1.50]
27. Jamil 2021 [40]	Retrospective cohort Pakistan	SARS-CoV-2	IMV: 33 CPAP: 28	No data	No data	No data	57.06 ± 12.63 53.75 ± 18.10	No data	- 0.69 [0.45, 1.05]
28. Myers 2023 [22]	Retrospective cohort USA	SARS-CoV-2	IMV: 2965 NIV: 2793	No data	No data	No data	60.8 ± 15.3 62.4 ± 12.7	No data	- 0.32 [0.30, 0.35]
29. Pasin 2022 [41]	Retrospective cohort USA	SARS-CoV-2	IMV: 141 CPAP: 141 CPAP-F: 89	No data	No data	4 (3–5) 4 (3–5)	68 (58–75) 66 (57–73)	114 (74–195) 120 (83–181) No data	- 0.61 [0.42, 0.89] 0.81 [0.55, 1.19]
30. Pia 2021 [42]	Retrospective cohort USA	SARS-CoV-2	IMV: 91 NIV: 131 NIV-F: 34	No data	No data	No data	67 (60–76) 67 (65–82) 69 (58–75)	No data	- 0.90 [0.78, 1.03] 1.02 [0.87, 1.20]
31. Rizwan 2022 [43]	Retrospective cohort Pakistan	SARS-CoV-2	IMV: 30 NIV: 44	No data	No data	No data	No data	No data	- 0.68 [0.53, 0.87]
32. Rocans 2022 [44]	Retrospective cohort Latvia	SARS-CoV-2	IMV: 156 CPAP: 374 CPAP-F: 235	No data	No data	No data	63 (62–63) 65 (63–66) 63 (61.0–65.4)	No data	- 0.91 [0.47, 1.09] 3.23 [0.81, 1.02]
33. Rajdev 2021 [45]	Retrospective cohort USA	SARS-CoV-2	IMV: 121 NIV: 232	TDV: 6.4 (0.5) FiO_2_: 93% PEEP: 9.28	No data	No data	No data	No data	- 0.15 [0.05, 0.44]
34. Wang 2020 [46]	Retrospective cohort China	SARS-CoV-2	IMV: 50 NIMV: 91	No data	No data	No data	64 (55–70) ^a^	108 (58–219) 261 (175–287)	- 0.42 [0.26, 0.68]
35. Zheng 2020 [47]	Retrospective cohort China	SARS-CoV-2	IMV: 15 HFNC: 19	No data	No data	No data	71 (60, 83) 66 (51, 72)	No data	Not estimable
36. Hamouri 2021 [48]	Case‒Control Jordan	SARS-CoV-2	IMV: 112 CPAP: 127	TDV: 6.18 PP: 29 PEEP: 16	No data	No data	67.7 ± 14.4 64.7 ± 12.8	No data	- Not estimable
37. Woo 2020 [50]	On trial – withdraw USA	SARS-CoV-2	IMV: no data SV: no data	No data	No data	No data	No data	No data	No data
38. Keith 2020 [51]	On trial – not recruiting USA	SARS-CoV-2	IMV: no data NIV: no data	No data	No data	No data	No data	No data	No data
39. Buddharaju 2021 [52]	Abstract congress USA	SARS-CoV-2	IMV: 35 NIMV: 23	No data	No data	No data	No data	No data	- 0.38 [0.14, 0.99]
40. Chowdhury 2021 [53]	Abstract congress USA	SARS-CoV-2	IMV: 49 HFNC: 125	No data	No data	No data	No data	No data	- 0.55 [0.30, 0.84]
41. Chang 2022 [54]	Abstract congress USA	SARS-CoV-2	IMV: 235 NIV: 398	No data	No data	No data	No data	No data	- 0.65 [0.51, 0.83]
42. Eswarappa 2021 [55]	Abstract congress UK	SARS-CoV-2	IMV: 80 CPAP: 215 CPAP-F: 31	No data	No data	No data	No data	No data	- 0.72 [0.54, 0.97] 1.36 [0.96, 1.92]
43. Flaatten 2021 [56]	Abstract congress Norway	SARS-CoV-2	IMV: 1777 NIV: 690 NIV-F: 331	No data	18.7 7.2 17	6.8 4.3 5.6	75.4 77.3 75.6	No data	- 1.66 [1.55, 1.78] 0.77 [0.65, 0.91]
44. Iftikhar 2021 [57]	Abstract congress UK	SARS-CoV-2	IMV: 34 CPAP: 95 CPAP-F: 24	No data	No data	No data	58.8 ± 13.2 61.7 ± 11.5 56 ± 12.8	No data	- 0.86 [0.49, 1.39] 1.98 [0.50, 1.92]
45. Mourisco 2022 [58]	Abstract congress Portugal	SARS-CoV-2	IMV: 47 NIV: 102	No data	No data	No data	70 ± 15 65 ± 15	99 ± 52 104 ± 66	- 0.89 [0.50, 1.57]
46. Napolitani 2021 [59]	Abstract congress Italy	SARS-CoV-2	IMV: 44 CPAP: 22	No data	No data	No data	No data	No data	- 0.85 [0.52, 1.37]
47. Yamamoto 2022 [60]	Abstract congress Japan	SARS-CoV-2	IMV: 1139 NIMV: 248 HFNC: 1072	No data	No data	No data	No data	No data	- 0.44 [0.30, 0.64] 0.28 [0.22, 0.36]

*IMV* invasive mechanical ventilation, *NIV* non-invasive ventilation, *NIMV* non-invasive mechanical ventilation, *HFNC* high flow nasal cannula, *CPAP* continuous positive airway pressure, *SV* splitter ventilator, *NIV-F* non-invasive ventilation failure, *NIMV-F* non-invasive mechanical ventilation failure, *HFNC-F* high flow nasal cannula failure, *CPAP-F* continuous positive airway pressure failure, *PEEP* positive end expiratory pressure (cm H_2_O), *PP* plateau pressure (cmH_2_O), *FiO*_*2*_ fraction of inspired oxygen, *TDV* tidal volume (ml/kg)

^a^No mean or median per group but general

### Risk of bias in individual studies

**Fig. 2:**
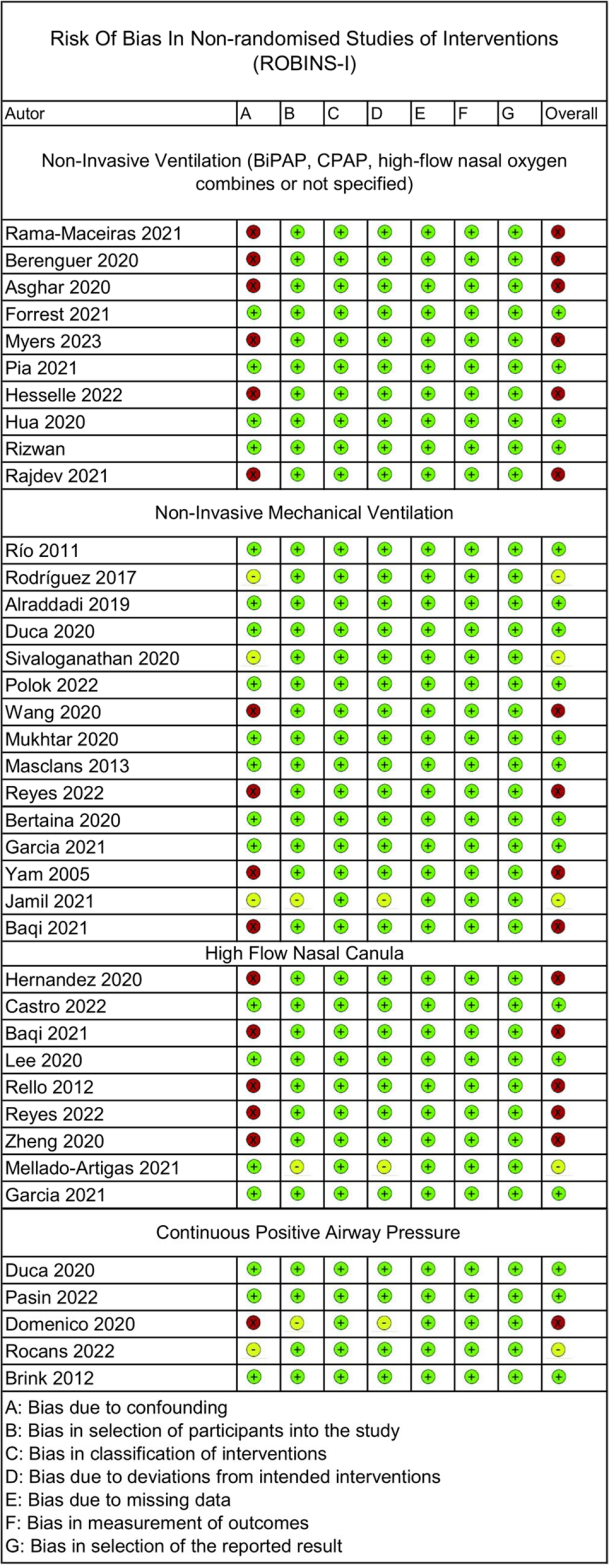
ROBINS-I evaluation for all ventilatory strategies

The analysis examined 36 NRS studies using the ROBINS-I since RCTs were absent. No bias assessment was performed for nine congress abstracts or two trial studies. Among the 16 NIMV studies, most had a low risk of bias due to missing data, in the measurement of outcomes and in the selection of the reported result, but only some overlooked critical risk factors such as age and disease severity. Nine HFNC studies were evaluated; although all had a low risk of bias, such as NIMV, some did not adjust for important risk factors. CPAP studies varied, with only a few having a low risk of bias in all domains. Additionally, eight studies lacking clear intervention distinctions were assessed. The details are shown in Fig. 2 and individual details for risk of bias assessments are reported in table S2.

### Syntheses of results for mortality

**Fig. 3:**
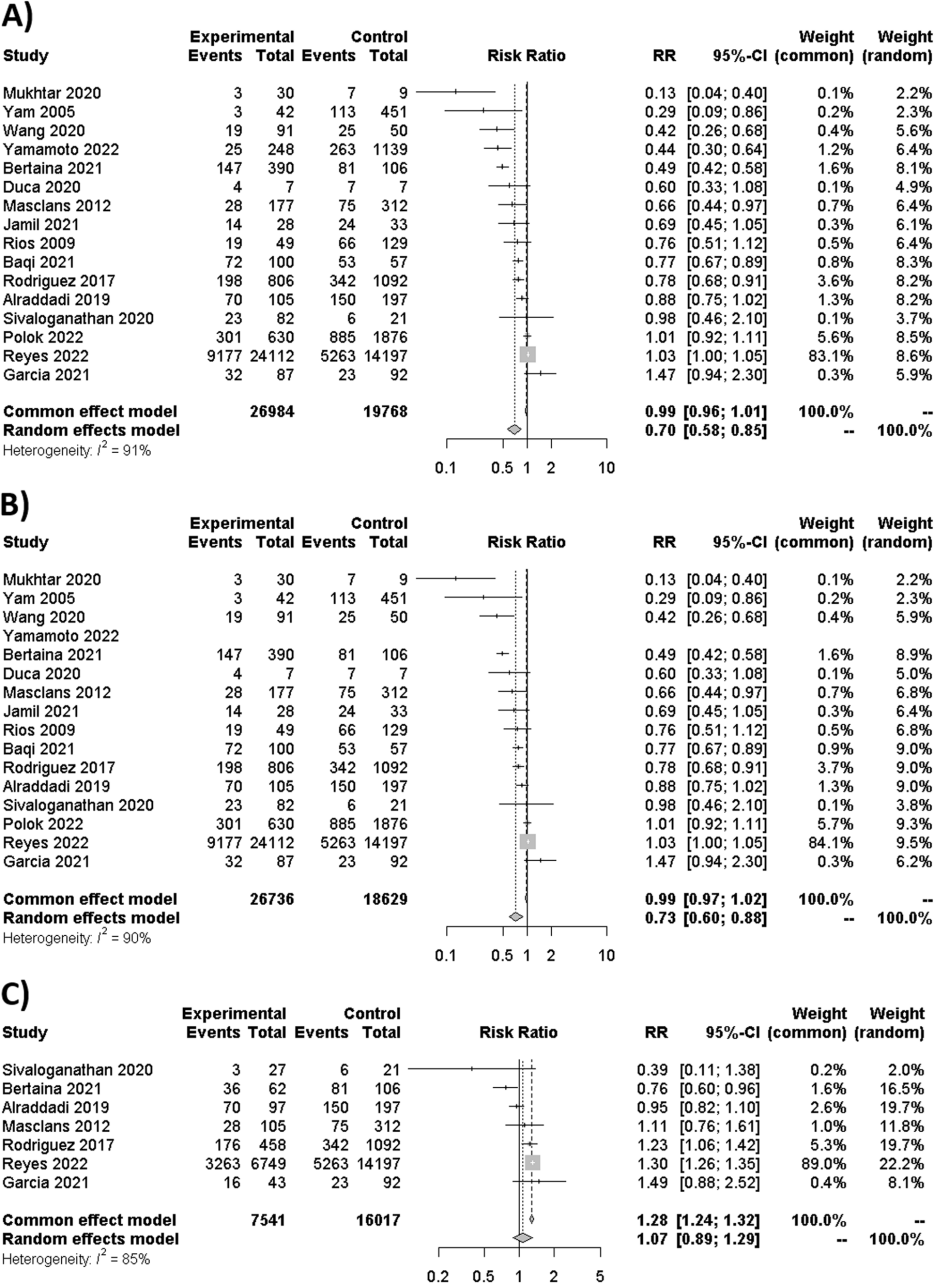
**A** Forest plot of the difference between NIMV and IMV for mortality with abstract congress. **B** Forest plot of the difference between NIMV and IMV for mortality without abstract congress. **C** Forest plot of the relationship between NIMV failure and IMV mortality

As no RCT was found, only NRS were considered for syntheses. In the comparison between NIMV and IMV, 15 studies and one abstract study revealed a statistically significant reduction in mortality, with an RR of 0.70 [95% CI 0.58–0.85; *I*^2^ = 91%] (Fig. 3A). By only considering 8 studies with the lowest bias for the sensitivity analysis, similar results were obtained with an RR of 0.72 [95% CI 0.52–0.99; *I*^2^ = 91%]. The same results were obtained when abstracts were excluded, with an RR of 0.73 [95% CI 0.60–0.80; *I*^2^ = 90%] (Fig. 3B). When assessing cases where NIMV failed and required subsequent intubation versus IMV, the analysis of seven studies revealed no significant differences in mortality (RR 1.07 [95% CI 0.89–1.29; *I*^2^ = 85%]) (Fig. 3C). Four studies with the lowest bias were considered for sensitivity analysis having similar results, with an RR of 0.97 [95% CI 0.78–1.21; *I*^2^ = 56%].

**Fig. 4:**
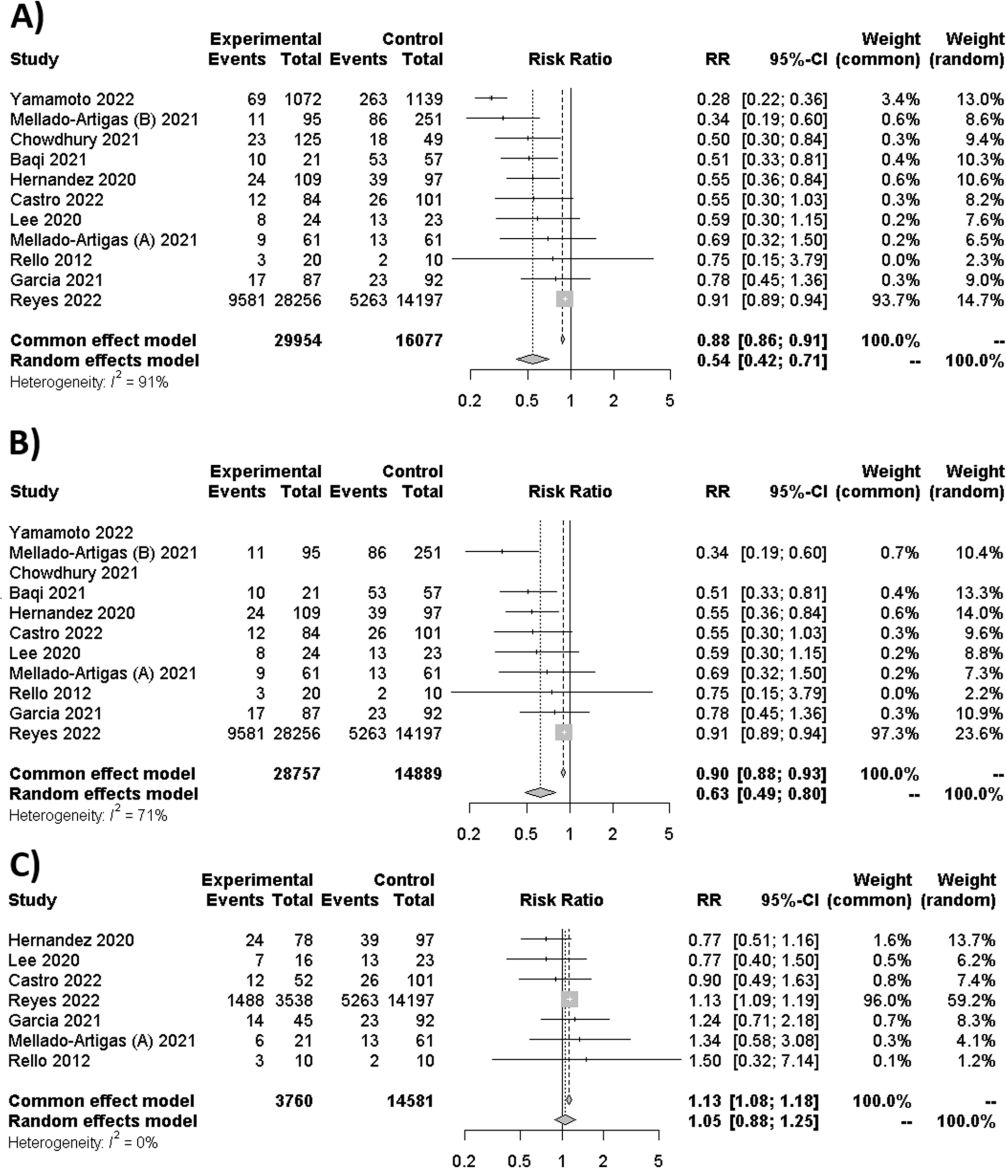
**A** Forest plot of HFNC therapy against IMV for mortality with an abstract congress. **B** Forest plot of HFNC therapy against IMV for mortality without an abstract congress. **C** Forest plot of HFNC failure versus IMV for mortality

For HFNC therapy compared to IMV, a meta-analysis of eight studies and two abstract congresses demonstrated a substantial decrease in mortality (RR 0.54 [95% CI 0.42–0.71; *I*^2^ = 91%]) (Fig. 4A). No differences were detected when abstracts were excluded (RR 0.63 [95% CI 0.49–0.80; *I*^2^ = 71%]) (Fig. 4B). Similar results for sensitivity analysis were obtained with 3 studies with the lowest bias risk (RR 0.65 [95% CI 0.46–0.92; *I*^2^ = 0%]). Conversely, when HFNC therapy failed and led to intubation versus IMV, seven studies revealed no statistically significant differences in mortality (OR 1.05 [95% CI 0.88–1.25; *I*^2^ = 0%]) (Fig. 4C). No differences were detected in the sensitivity analysis of mortality among the 3 studies with the lowest risk of bias (RR 0.98 [95% CI 0.69–1.38; *I*^2^ = 0%]).

**Fig. 5:**
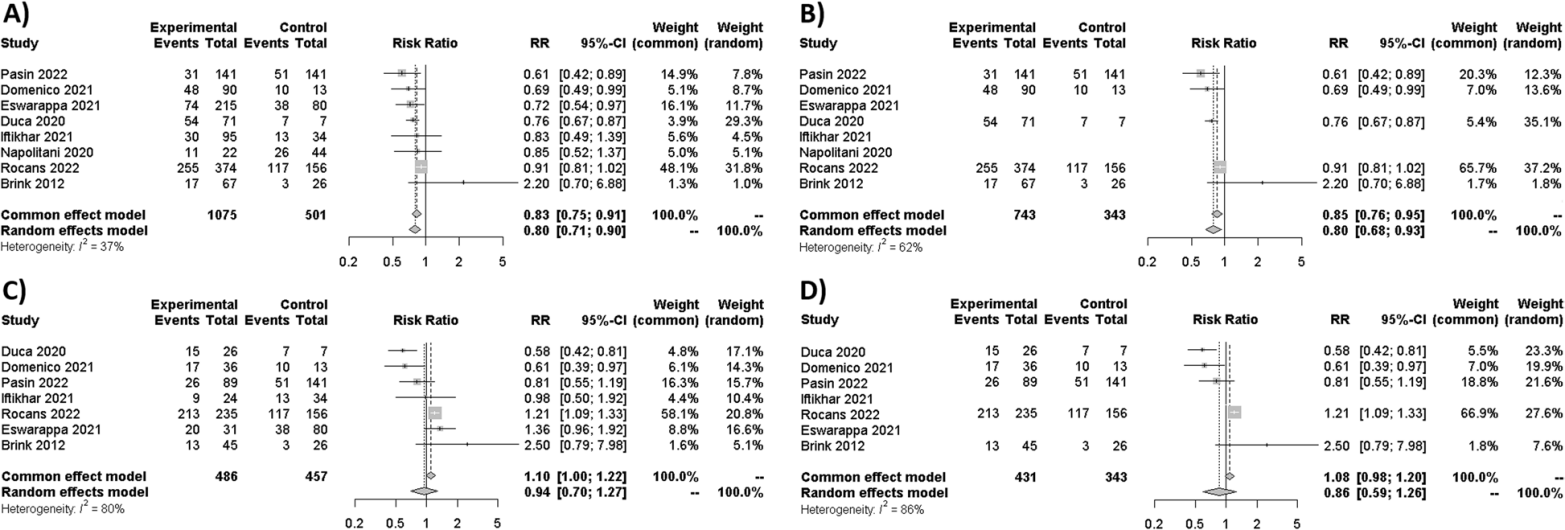
**A** Forest plot of the association between CPAP and IMV in terms of mortality with abstract congress. **B** Forest plot of the association between CPAP and IMV for mortality without an abstract congress. **C** Forest plot of mortality in patients who experienced CPAP failure versus those who experienced IMV with abstract congress. **D** Forest plot of mortality without abstract congress in patients who experienced CPAP failure versus those who experienced IMV

Regarding CPAP, a meta-analysis of five studies and three abstracts revealed a reduction in mortality (RR 0.80 [95% CI 0.71–0.90; *I*^2^ = 37%]) (Fig. 5A). No changes were identified when abstracts were excluded (RR 0.80 [95% CI 0.68–0.93; *I*^2^ = 62%]) (Fig. 5B). The same results were obtained using only two studies with the lowest risk of bias for the sensitivity analysis (RR 0.73 [95% CI: 0.62–0.87; *I*^2^ = 18%]). In cases where CPAP failed and resulted in intubation versus IMV, the analysis of seven studies and two abstract congresses demonstrated an increase in mortality, although the confidence interval was wider (RR 0.94 [95% CI 0.70–1.27; *I*^2^ = 80%]) (Fig. 5C), with no differences if abstract congress was excluded (RR 0.86 [95% CI: 0.56–1.26; *I*^2^ = 86%]) (Fig. 5D). Important differences were identified for the sensitivity analysis using two studies with the lowest risk of bias (RR 0.67 [95% CI 0.49–0.92; *I*^2^ = 36%]).

Calculated unadjusted RR for each study used in the meta-analysis can be found in Figs. 3A–C, 4A–C, and 5A–D, while adjusted RR reported by the corresponding authors in the included studies can be found in table S1.

### Syntheses of results for LOS and other outcomes

**Fig. 6:**
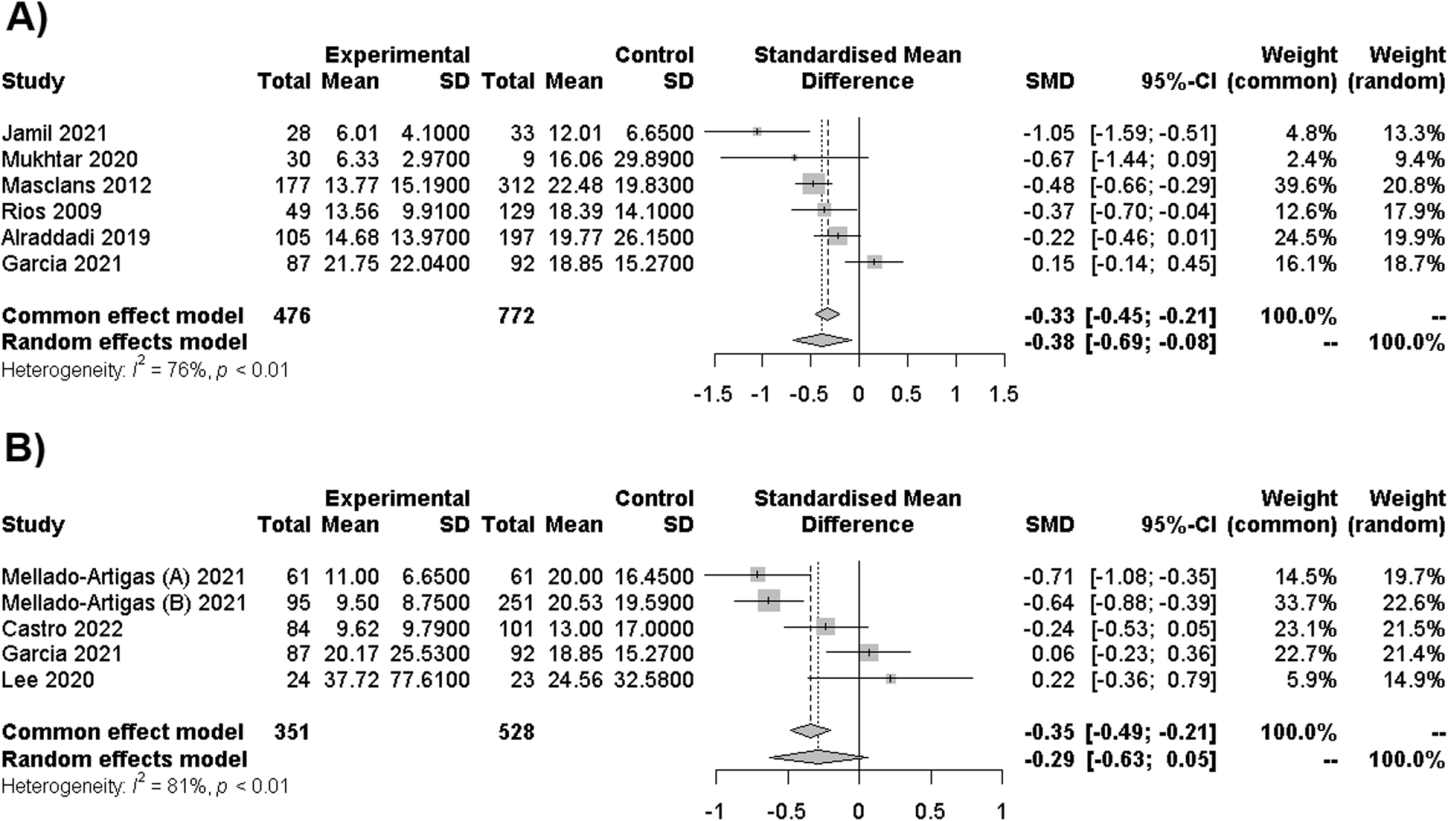
Forest plot of LOS days in ICU. **A** Forest plot of the association between NIMV and IMV in terms of LOS days in ICU. **B** Forest plot of the association between HFNC and IMV for LOS days in ICU

In the comparison between NIMV and IMV, 6 NRS and no abstract congress revealed a statistically significant reduction in LOS mean, with a difference of − 0.38 days [95% CI − 0.69: − 0.08; *I*^2^ = 76%] (Fig. 6A). By considering only 5 studies with the lowest risk of bias for sensitivity analysis, the LOS mean difference was the same with − 0.27 days [95% CI − 0.52: − 0.06; *I*^2^ = 71%]. In the case of HFNC against IMV with 4 NRS and no abstract congress, no significant differences were identified as mean difference was − 0.29 days [95% CI − 0.64: 0.06; *I*^2^ = 81%] (Fig. 6B). Different results were obtained when only including studies with the lowest bias risk with a mean difference of 0.98 days [95% CI 0.69: 1.38; *I*^2^ = 0%] higher. No meta-analysis for CPAP was possible as no study report LOS days in ICU, as same goes to NIMV-failure and HFNC-failure as no study report LOS days exclusive for those scenarios. In the same way, not enough studies with the same ventilation strategy reported nosocomial infection or barotrauma to evaluate them in a meta-analysis. In the case of nosocomial infection, Masclans [20] report 60 (19.2%) cases in IMV and 20 (11.1%) cases for NIMV, while Wang [46] reported 14 (28.0%) cases for IMV and 15 (16.5%) for NIMV. For barotrauma, Brink [19] reported 4 (15%) cases in IMV and 4 (6%) cases in CPAP, while Hamouri [48] reported 17 (33.3%) cases in IMV and 34 (66.7%) cases in NIV.

### Reporting biases

**Fig. 7:**
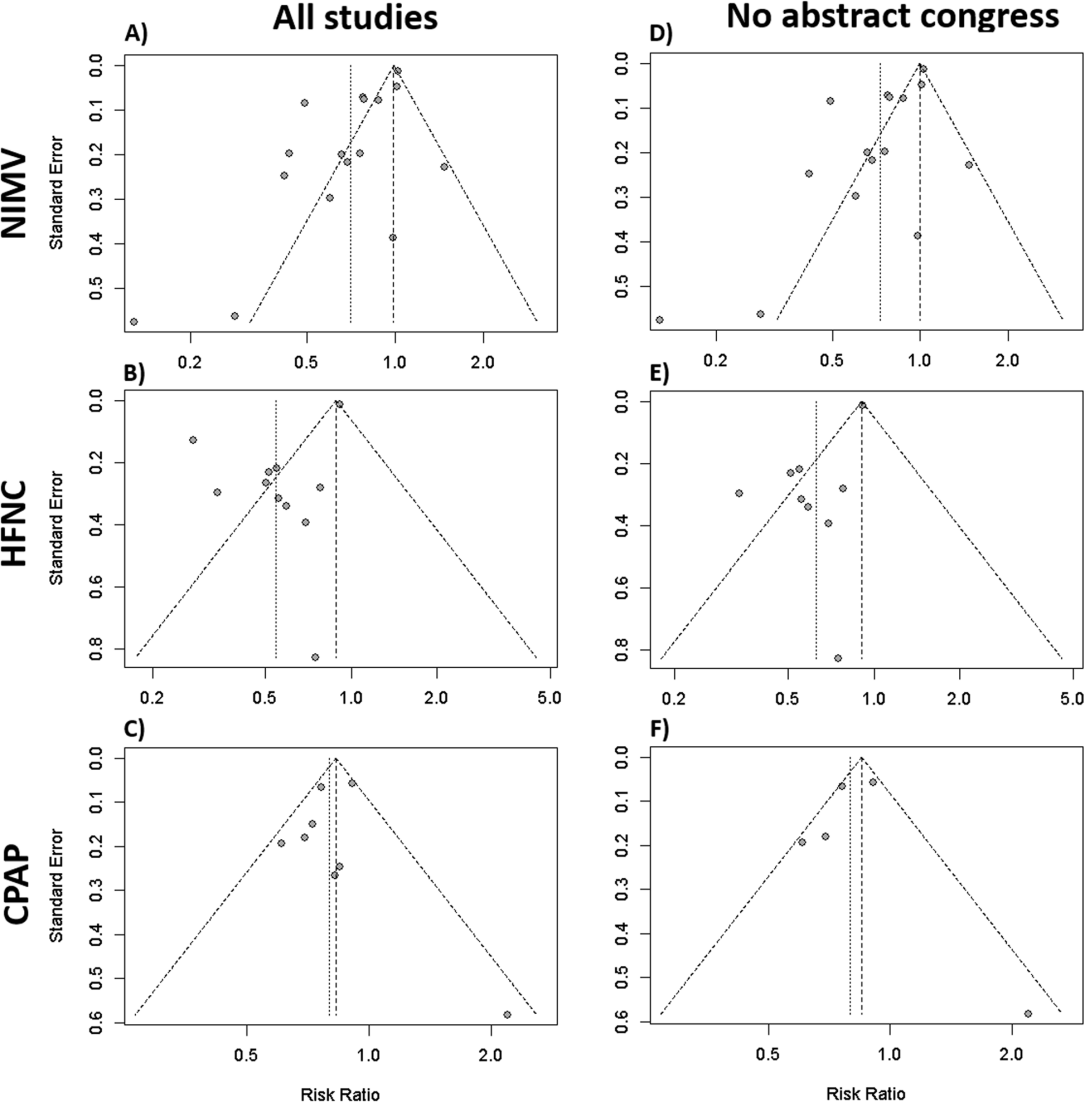
**A** Funnel plot of the difference between NIMV and IMV for mortality with abstract congress. **B** Funnel plot of the difference between NIMV and IMV for mortality without abstract congress. **C** Funnel plot of HFNC therapy against IMV for mortality with an abstract congress. **D** Funnel plot of HFNC therapy against IMV for mortalitywithout an abstract congress. **E** Funnel plot of the association between CPAP and IMV mortality with abstract congress. **F** Funnel plot of the association between CPAP and IMV for mortality without an abstract congress

**Fig. 8:**
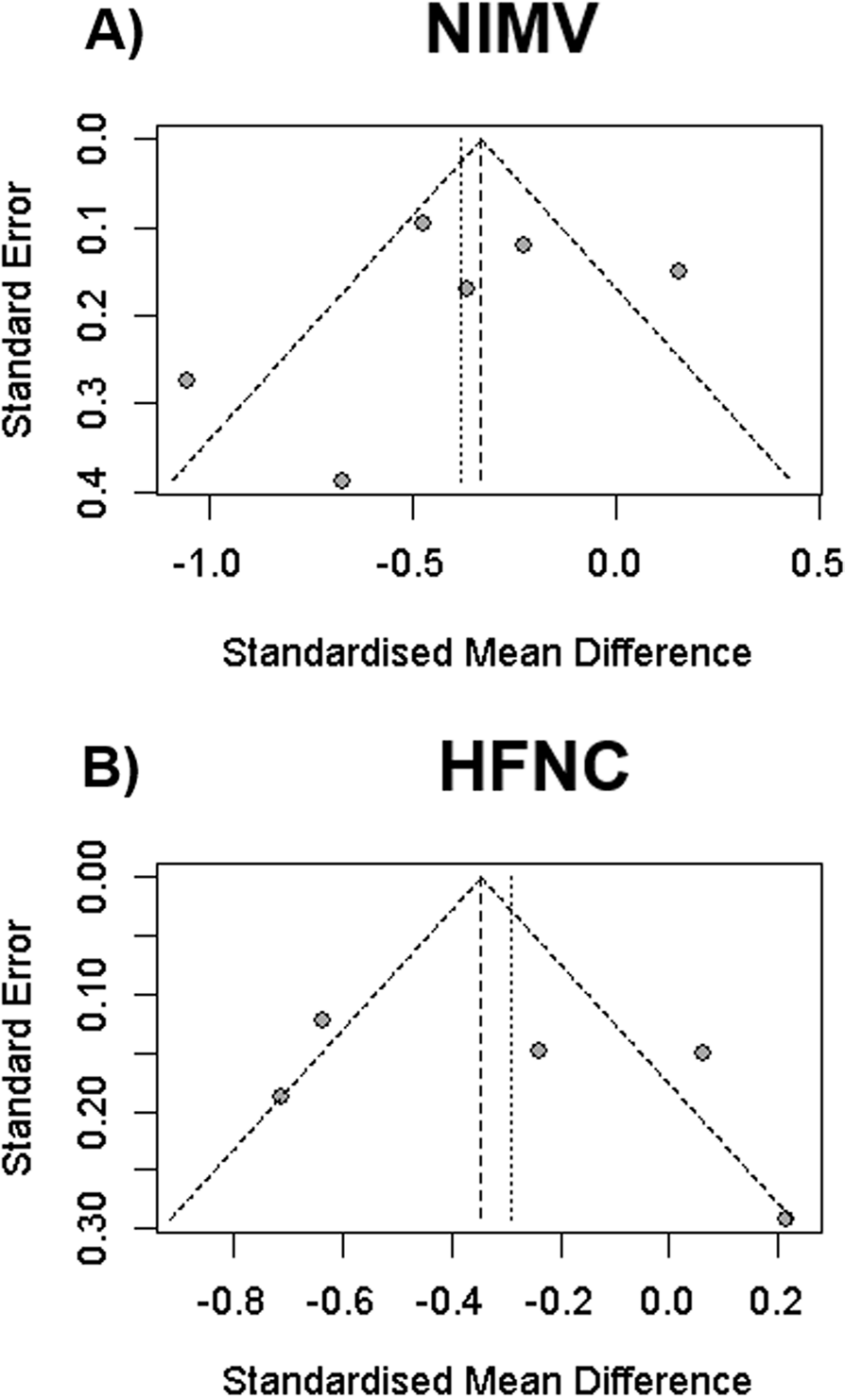
**A** Funnel plot of the difference between NIMV and IMV for LOS days in ICU. **B** Funnel plot of the difference between NIMV and HFNC for OS days in ICU

For the studies and abstracts that involved NIMV or HFNC therapy in mortality, asymmetry and risk of bias were identified against the intervention in their funnel plots (Fig. 7A, B). Different results were obtained for studies that involved CPAP, as asymmetry was not observed in their funnel plot (Fig. 7C). Similar results were obtained via Egger’s test, with NIMV showing a β0 coefficient of − 2.56 and HFNC exhibiting a β0 coefficient of − 2.54. For CPAP, Egger’s test was not possible because fewer than 10 studies were included. No differences were detected when abstracts were excluded (Fig. 7D–F). In LOS days in ICU, no significant asymmetry was observed in the funnel plots for NIMV and HFNC (Fig. 8A, B), but no Egger’s test was possible as the number of studies was too small to test for small study effects.

### Meta-regression and sensitivity analyses

In the meta-regression analyses comparing different noninvasive ventilation (NIV) modalities with invasive mechanical ventilation (IMV) for mortality, specific factors were considered to reduce heterogeneity. For NIMV versus IMV, models that included age, PaO_2_/FiO_2_, and the specific virus type significantly reduced heterogeneity. The model excluding imputed data and abstracts proved most effective. In the case of NIMV failure versus IMV, models incorporating SOFA score and either age or PaO_2_/FiO_2_ (depending on imputed data usage) reduced heterogeneity to zero. Notably, only the model with imputed data showed statistically significant covariates. For HFNC therapy versus IMV, all models utilized the SOFA score as a covariate, with some including age and APACHE II score. These models achieved zero heterogeneity, with the SOFA score being statistically significant in the model without imputed data or abstract congresses. Similarly, for HFNC failure versus IMV, the APACHE II score and, in some cases, age were used as covariates. All models achieved zero heterogeneity, with the APACHE II score being statistically significant in the model using imputed data. In the meta-regression analysis of the correlation between CPAP and IMV, age and PaO_2_/FiO_2_ were used, and zero heterogeneity was achieved across all the models. Age was statistically significant in the two models, while PaO_2_/FiO_2_ was significant in all CPAP-failure models. The detailed results of these analyses can be found in Tables 2 and 3.

**Table 2:** NIV modalities against IMV for mortality Meta-regressions models

NIV modalities and model	*n* studies	*I*^2^	R^2^	Covariant	Estimate
NIMV **with** imputed data and abstract congress	17	92.98	0	Intercept	− 0.52
				Influenza	0.11
				Age	0.006
				PaO_2_/FiO_2_	− 0.001
NIMV **without** imputed data and **no** abstract congress	8	85.56	0	Intercept	11.76
				Influenza	− 3.21
				Age	0.006
				PaO_2_/FiO_2_	− 0.17
NIMV **with** imputed data and **no** abstract congress	15	93.19	17.47	Intercept	− 0.52
				Influenza	0.11
				Age	0.006
				PaO_2_/FiO_2_	− 0.001
HFNC **with** imputed data and abstract congress	11	69.61	14.3	intercept	− 2.097
				Age	0.01
				SOFA	0.19
HFNC **without** imputed data and **no** abstract congress	5	9.01	69.45	intercept	− 15.04
				APACHEII	1.18
				SOFA	0.77
HFNC **with** imputed data and **no** abstract congress	9	36.23	50.02	intercept	− 2.94
				Age	0.02
				SOFA	0.19
CPAP **with** imputed data and abstract congress	8	0	100	Intercept	0.41
				Age	− 0.01
				PaO_2_/FiO_2_	− 0.01
CPAP **without** imputed data and abstract congress	6	42.43	0	Intercept	0.81
				Age	− 0.02
CPAP **with** imputed data and **no** abstract congress	5	57.90	0	Intercept	0.96
				Age	− 0.02

**Table 3:** NIV failure against IMV for mortality

Model	*n* studies	*I*^2^	R^2^	Covariant	Estimate
NIMV failure **with** imputed data and **no** abstract congress	7	12.11	97.01	Intercept	1.15
				PaO_2_/FiO_2_	− 0.004
				SOFA	− 0.06
NIMV failure **without** imputed data and **no** abstract congress	5	0	100	Intercept	− 0.05
				SOFA	− 0.15
				Age	0.01
HFNC failure **with** imputed data and **no** abstract congress	7	0	100	Intercept	8.02
				APACHEII	− 0.61
				Age	− 0.02
HFNC failure **without** imputed data and **no** abstract congress	5	0	0	Intercept	8.06
				APACHEII	− 0.72
CPAP failure **with** imputed data and abstract congress	7	0	100	Intercept	3.81
				PaO_2_/FiO_2_	− 0.03
				Age	− 0.04
CPAP **without** imputed data and abstract congress	4	36.45	0	Intercept	4.14
				PaO_2_/FiO_2_	− 0.01
				Age	− 0.4
CPAP **with** imputed data and **no** abstract congress	5	0	100	Intercept	3.71
				PaO_2_/FiO_2_	− 0.01
				Age	− 0.04

In the case for LOS days in ICU, no model could reduce heterogeneity below 50% in NIMV. The best model presents an *I*^2^ of 50.70% with imputed data from APACHE II, virus, and age. Without imputed data and 5 studies, only PaO_2_/FiO_2_ can reduce *I*^2^ of 50.90%. Different results were obtained for HFNC with imputed data, in which the inclusion of PaO_2_/FiO_2_, APACHE II, and Age can reduce heterogeneity to 0%. Without imputed data, PaO_2_/FiO_2_ and APACHE II continue to reduce heterogeneity near to 0%. Models are detailed in Table 4.

**Table 4:** NIV against IMV for LOS days in ICU

Model	*n* studies	*I*^2^	R^2^	Covariant	Estimate
NIMV **with** imputed data	6	50.77	70	Intercept	− 13.50
				APACHEII	0.12
				Virus	2.81
				Age	0.18
NIMV **without** imputed data	5	50.98	79.41	Intercept	0.42
				PaO_2_/FiO_2_	− 0.004
HFNC **with** imputed data	4	0	100	Intercept	− 12.81
				SOFA	0.22
				Age	0.19
				PaO_2_/FiO_2_	− 0.01
HFNC **without** imputed data	3	3.64	99.52	Intercept	− 5.83
				SOFA	− 0.20
				PaO_2_/FiO_2_	0.03

### Quality of evidence

Although only NRS were used, all outcomes and interventions start with a high (⨁⨁⨁⨁) certainty of evidence given due to the ROBINS-I tool being used. For the risk of bias, the concerns about comparability bias were prevalent due to insufficient adjustments for crucial factors such as age or PaO_2_/FiO_2_ in many studies, leading to lowering the GRADE to medium (⨁⨁⨁◯) in NIMV and HFNC therapy followed by intubation and low (⨁⨁◯◯) for the rest of the interventions in mortality. Finally, due to serious imprecision for NIMV, GRADE was lowered to low (⨁⨁◯◯). No problems were detected in inconsistency, indirectness, or imprecision for HFNC therapy followed by intubation. NIMV failure presents serious concerns in indirectness and imprecision, sow certainty was very low (⨁◯◯◯). In the case of LOS days in ICU, NIMV was lowered to medium due to no meta-regression model which could reduce heterogeneity, while HFNC having sensitivity analysis with a different result also lowered the certainty to medium. For a detailed overview, refer to Table 5.

**Table 5:** GRADE evaluation for noninvasive ventilation compared with invasive mechanical ventilation for ARF due to viral pneumonia (assessed with ROBINS-I)

Intervention	Certainty assessment							№ of patients		Certainty	Importance
	№ of studies	Study design	Risk of bias	Inconsistency	Indirectness	Imprecision	Other considerations	NIV	IMV		
Mortality (any cause)											
NIMV	16	Observational studies	Serious^a^	Not serious	Not serious	Serious^b^	All plausible residual confounding would reduce the demonstrated effect	10,121/26,992 (37.5%)	7396/19,782 (37.4%)	⨁⨁◯◯ Low	CRITIC
NIMV-F	7	Observational studies	Very serious^c^	Very serious^d^	Not serious	Very serious^e^	All plausible residual confounding would reduce the demonstrated effect	3628/7741 (46.9%)	5940/16,017 (37.1%)	⨁◯◯◯ Very low	CRITIC
HFNC	10	Observational studies	Very serious^f^	Not serious	Not serious	Not serious	All plausible residual confounding would reduce the demonstrated effect	9761/29,952 (32.6%)	5845/16,138 (36.2%)	⨁⨁◯◯ Low	CRITIC
HFNC-F	7	Observational studies	Serious^g^	Not serious	Not serious	Not serious	All plausible residual confounding would reduce the demonstrated effect	1554/3760 (41.3%)	5379/14,581 (36.9%)	⨁⨁⨁◯ Medium	CRITICAL
CPAP	8	Observational studies	Very serious^h^	Not serious	Not serious	Not serious	All plausible residual confounding would reduce the demonstrated effect	532/1178 (45.2%)	265/501 (52.9%)	⨁⨁◯◯ Low	CRITIC
CPAP-F	7	Observational studies	Very serious^i^	Very serious^j^	Not serious	Not serious	All plausible residual confounding would reduce the demonstrated effect	313/486 (64.4%)	239/457 (52.2%)	⨁◯◯◯ Very low	CRITIC
Length of stay in ICU days											
NIMV	6	Observational studies	Not serious	Not serious	Not serious	Serious^k^	All plausible residual confounding would reduce the demonstrated effect	476	772	⨁⨁⨁◯ Medium	IMPORTANT
HFNC	4	Observational studies	Not serious	Serious^l^	Not serious	Not serious	All plausible residual confounding would reduce the demonstrated effect	351	528	⨁⨁⨁◯ Medium	IMPORTANT

**Explanations**

a. 8 of the 16 studies used did not adjust or assess important differences in the choice of cohort

b. No meta regression model could reduce heterogeneity

c. Only 2 of the 7 studies adjusted for or assessed important differences in the choice of cohort. e. 3 of the 7 studies have different effect directions

d. The different magnitude of the effect even in the studies that have the same effect direction

e. 3 of the 7 studies used did not adjust or assess important differences in the choice of cohort

f. Only one study did not adjust or assess important differences in the choice of cohort

g. 4 of the 7 studies used did not adjust or assess important differences in the choice of cohort

h. 2 of 8 studies used did not adjust or assess important differences in the choice of cohort

i. 2 of 7 studies used did not adjust or assess important differences in the choice of cohort

j. The different magnitude of the effect even in the studies that have the same effect direction

k. 2 of the 6 studies used did not adjust or assess important differences in the choice of cohort

l. The sensitivity analysis yields a different result

## Discussion

In this systematic review of patients with virus-induced ARF, most of the studies comparing HFNC, CPAP, and NIV to IMV showed improved survival or no difference in ICU mortality and LOS days in ICU. These results can be contrasted by the exploratory meta-analysis, as their association coefficients revealed significant reductions in mortality or LOS days in ICU. For all NIV-failure patients (NIMV, HFNC, or CPAP), most of the studies did not show an increase in mortality, and similar results were shown in their exploratory meta-analysis. Exploratory meta-regression also explored and explained heterogeneity in all ventilatory strategies, indicating that the severity of the patients affected the results. For LOS days in ICU, only NIMV ventilation reduce those days while HFNC cannot. These results provide valuable insights into the efficacy of different ventilation strategies in managing ARF due to viral infections.

Four meta-analyses and systematic reviews [5, 61–63] have explored the correlation between NIMV as a ventilatory strategy and its impact on mortality. However, our study differs from these previous works in several key aspects. First, the clinical settings assessed varied among these studies. While three of them focused on patients with acute hypoxemic respiratory failure resulting from diverse etiologies, each employing different definitions [61–63], one study specifically examined patients with acute hypoxemic respiratory failure caused by COVID-19, SARS, and MERS [5]. In contrast, our study focused solely on patients with ARF caused by COVID-19, SARS, MERS, or H1N1. Second, the types of studies included in the analysis also differed. Two meta-analyses incorporated only randomized controlled trials (RCTs) [61, 63], while the others used various study designs. In the present study, congress abstracts were included without bias due to insufficient information on their methodology. Consequently, meta-regression models were constructed with and without these abstracts, revealing variations in *I*^2^ and R^2^ across all NIV modalities. Third, the methods of analysis varied across the studies. Two of them employed pairwise meta-analysis [61, 62], one utilized a network meta-analysis [63], and one, like our study, solely conducted a systematic review [5]. The latter study included several types of interventions [5], while the other two studies assessed only helmet ventilation as an intervention [62]. Additionally, the types of interventions evaluated were not uniform in these studies.

The reduction in mortality observed in NIMV can be attributed to several factors. First, IMV often requires additional interventions such as sedation and vasopressors, which may contribute to higher mortality rates, particularly in critically ill patients with prolonged ICU stays. Additionally, IMV is associated with an increased risk of nosocomial infections, such as ventilator-associated pneumonia [64], making noninvasive ventilation strategies more advantageous for mitigating these complications [65]. However, caution is required when interpreting the findings of studies comparing NIMV failure followed by intubation to initial IMV, as some studies indicate no reduction in mortality for patients requiring intubation after NIMV failure. Further research is needed to identify specific patient factors contributing to increased mortality risk and to refine recommendations for NIMV management.

In the case of HFNC therapy, the positive outcomes in the included studies could be attributed to its ability to generate a low level of positive pressure in the upper airway, possibly improving oxygenation [66]. While some patients who experience HFNC failure have increased mortality, the ROX index has been found to be suitable for predicting HFNC failure in COVID-19 patients with acute hypoxemic respiratory failure [67]. However, many studies in the review did not incorporate the ROX index, and future studies comparing HFNC therapy against IMV should consider its inclusion to improve patient selection accuracy.

Compared with IMV, CPAP was associated with a reduction in mortality in patients with viral-induced ARF. CPAP increases airway pressure, ameliorates arterial oxygenation, increases end-expiratory lung volume, and improves cardiac function [4]. Patients with a PaO_2_/FiO_2_ ratio ranging from 100 to 200 can benefit from CPAP, as studies including patients within this range reported positive outcomes. However, due to the limited number of available studies, the safety profile of CPAP in patients with ARF of viral origin requires further investigation.

One of the reasons for the reduction on LOS days in ICU by NIMV and not in HFNC may be attributable to the positive end expiratory pressure (PEEP). The PEEP can improve ventilation-perfusion (VQ) mismatches [68], and those advantages can be seen in less mortality and less LOS days in ICU. Similarly, not having an invasive device may result in fewer infections which translate into fewer LOS days in ICU and less mortality. However, this last hypothesis was not evaluated in the present review with meta-analysis since there were not enough studies reporting nosocomial infections such as ventilator-associated pneumonia.

There are several strengths focused on the search strategy. An example of this was the inclusion of search engines such as Trials.gov and regulatory agencies of Latin American countries for gray literature, which allowed us to reduce the risk of publication bias. However, asymmetries were identified both by funnel plot and by Egger’s test, but there was a bias toward publishing studies with negative results; nevertheless, favorable results were found for all NIV modalities. This tells us that the problem is not in the lack of studies but in their quality with incomplete information.

Our study is subject to several limitations. First, this study relied solely on observational studies due to logistical and ethical challenges in conducting clinical trials during the early stages of the COVID-19 pandemic, potentially introducing biases and overestimating effects. This constraint is reflected in the review’s low to medium GRADE recommendations. For example, a meta-analysis by Masaaki Sakuray comparing NIMV to IMV in ARDS patients yielded mixed results depending on the device used. Additionally, extracting ICU stay data as medians with interquartile ranges posed challenges due to heterogeneity among patients, making assumptions for conversion into means with standard deviations inappropriate. The wide variance in patient numbers across studies also significantly impacts heterogeneity, as observed in sensitivity analyses for each NIV modality. Furthermore, the lack of distinction between ICU and hospital mortality in some studies required an assumption that all deaths occurred in the ICU if not specified.

Moreover, the use of random effects models was necessary due to anticipated high heterogeneity among critical patients, but this limits the generalizability of the meta-analysis results. Subgroup analysis could mitigate this limitation, but defining cutoff points for continuous covariates may introduce biases. Additionally, the absence of mean PaFiO_2_ ratios in most studies hinders the ability to draw definitive conclusions on the efficacy of NIV or HFNC therapy in ARF patients.

Furthermore, the inclusion of abstracts and missing data limited the possibility of inferential analysis. The imputed covariate data, such as age, PaO_2_/FiO_2_, SOFA score, and APACHE II score, were explored in meta-regression models against studies that reported those covariates, along with abstract congress results. However, due to the limitations mentioned, no clinical recommendations can be made based on these meta-regressions. Although imputed data may reduce variance in observational studies, the multitude of NIV modalities could counteract this effect. To address this, data imputation was stratified based on NIV modality. Recommendations for critical care research include the need to report covariates used in meta-regression models. Abstract congresses, due to being on trial, can present different results once they are published. To prevent this bias, multiple meta-analyses and meta-regressions with and without abstract congress were performed, which were used for sensitivity analysis. However, future updates to the present systemic review will be necessary once these abstract conferences are reviewed and published for a more complete and precise evaluation.

## Conclusion

The findings of this systematic review support the use of alternative noninvasive oxygenation and ventilation strategies as viable alternatives to conventional respiratory ventilation for managing viral-induced ARF. Although it is essential to interpret these findings with caution given the overall low to medium certainty of the evidence and the quality of being an exploratory meta-analysis, the integration of these modalities as part of the management strategies of these patients could help reduce the utilization of ICU beds, invasive ventilators, and costs in both developed and developing countries.

## Supplementary Information

Additional file 1: Table S1 can be found at Study Summary COVID-19 ventilation.

Additional file 2: Table S2 can be found at TableS2.

## Data Availability

The datasets used and/or analyzed during the current study are available from the corresponding author upon reasonable request. The data from the extracted studies are available in the supplementary data, and the analyses are in R code.
